# Finding the dark matter: Large language model‐based enzyme kinetic data extractor and its validation

**DOI:** 10.1002/pro.70251

**Published:** 2025-08-15

**Authors:** Galen Wei, Xinchun Ran, Runeem AI‐Abssi, Zhongyue Yang

**Affiliations:** ^1^ Department of Chemistry Vanderbilt University Nashville Tennessee USA; ^2^ Center for Structural Biology Vanderbilt University Nashville Tennessee USA; ^3^ Vanderbilt Institute of Chemical Biology Vanderbilt University Nashville Tennessee USA; ^4^ Data Science Institute Vanderbilt University Nashville Tennessee USA; ^5^ Department of Chemical and Biomolecular Engineering Vanderbilt University Nashville Tennessee USA

**Keywords:** deep learning, enzymology data, kinetics parameter, large language model

## Abstract

Despite the vast number of enzymatic kinetic measurements reported across decades of biochemical literature, the majority of relational enzyme kinetic data—linking amino acid sequence, substrate identity, kinetic parameters, and assay conditions—remains uncollected and inaccessible in structured form. This constitutes a significant portion of the “dark matter” of enzymology. Unlocking these hidden data through automated extraction offers an opportunity to expand enzyme dataset diversity and size, critical for building accurate, generalizable models that drive predictive enzyme engineering. To address this limitation, we built EnzyExtract, a large language model‐powered pipeline that automates the extraction, verification, and structuring of enzyme kinetics data from scientific literature. By processing 137,892 full‐text publications (PDF/XML), EnzyExtract collected more than 218,095 enzyme–substrate–kinetics entries, including 218,095 *k*_cat_ and 167,794 *K*_*m*_ values. These entries are mapped to enzymes spanning 3569 unique four‐digit EC numbers, with a total of 84,464 entries assigned at least a first‐digit EC number. EnzyExtract identified 89,544 unique kinetic entries (*k*_cat_ and *K*_*m*_ combined) absent from BRENDA, significantly expanding the known enzymology dataset. The newly curated dataset was compiled into a database named EnzyExtractDB. EnzyExtract demonstrates high accuracy when benchmarked against manually curated datasets and strong consistency with BRENDA‐derived data. To create model‐ready datasets, enzyme and substrate sequences were aligned to UniProt and PubChem, yielding 92,286 high‐confidence, sequence‐mapped kinetic entries. To assess the practical utility of our dataset, we retrained several state‐of‐the‐art *k*_cat_ predictors (including MESI, DLKcat, and TurNuP) using EnzyExtractDB. Across held‐out test sets, all models demonstrate improved predictive performance in terms of RMSE, MAE, and *R* ^2^, highlighting the value of high‐quality, large‐scale, literature‐derived EnzyExtractDB for enhancing predictive modeling of enzyme kinetics. The EnzyExtract source code and the database are openly available at https://github.com/ChemBioHTP/EnzyExtract, and an interactive demo can be accessed via Google Colab at https://colab.research.google.com/drive/1MwKSEZzLPNOseksRshbzkkFoO_cgJhva.

## 1 INTRODUCTION

Enzyme kinetic data are essential for uncovering the catalytic origin of enzymatic function and evolution (Markin et al., 2021). They serve as the foundation for training predictive AI models of enzymatic rate constant and specificity constant (Kroll et al., 2023). While thousands of scientific publications report detailed enzyme kinetic data each year, the vast majority of this critical information—enzyme kinetic parameters linked to sequence, substrate, and experimental conditions—remains scattered throughout the literature, essentially forming what we call the “dark matter” of enzymology data. The gap between published knowledge and structured, machine‐readable data poses a major obstacle to advancing quantitative modeling across biochemistry, computational enzyme engineering, metabolic engineering, and systems biology (Jurich et al., 2025; Yang et al., 2023).

Traditional approaches to enzyme data curation rely on painstaking manual extraction by expert curators, which remains essential for capturing enzyme function knowledge in FAIR open knowledgebases. However, these efforts simply cannot keep pace with the exponential growth of new discoveries and publications. Despite the existence of BRENDA (Schomburg et al., 2002) and SABIO‐RK (Wittig et al., 2012) for enzyme kinetic data storage, databases capture only a fraction of the published kinetic parameters, leaving a wealth of sequence–function–condition relationships unexplored. Specifically, in 2024 alone, over 19,700 articles containing the keyword “enzyme kinetics” were published (searched using Google Scholar), and more than 200,000 related publications have accumulated over the past two decades. The vast reservoir of kinetic data is still locked in the literature, far exceeding the size of any existing well‐curated kinetic database. Standardization initiatives such as EnzymeML (Lauterbach et al., 2023) offer promising frameworks for the structured reporting and exchange of enzymatic data; however, their adoption by the broader experimental community remains limited, and much of the available data continues to exist in unstructured formats. This persistent lack of accessible, high‐quality kinetic datasets constitutes a significant bottleneck for the development of data‐driven approaches, particularly for machine learning models that require large, diverse, and well‐annotated datasets.

Recent efforts have emerged that leverage AI‐assisted approaches to extract enzymology data from literature. FuncFetch introduced a large language model (LLM)‐assisted workflow that integrates multiple tools with GPT‐4 to screen thousands of manuscripts for enzyme‐substrate interactions (Smith et al., 2025). When applied to plant enzyme families, FuncFetch was used to screen over 26,000 papers and extract qualitative information about enzyme activities from over 5400 selected papers. Similarly, EnzChemRED (Lai et al., 2024) was developed to collect a specialized dataset of 1210 expert‐curated abstracts with annotated enzymes and chemical reactions using identifiers from UniProtKB and ChEBI. However, neither tool can extract quantitative kinetic parameters of enzyme catalysis. The development of enzyme Co‐Scientist (Jiang et al., 2025) from Zhejiang lab represents meaningful progress, extracting over 91,000 *k*_cat_ and *K*_*m*_ records from the literature, but this dataset cannot be readily used for predictive modeling due to the lack of mapping to protein sequences. This highlights a critical gap in current approaches: the need for comprehensive extraction of quantitative kinetic data tied to specific sequences. Meanwhile, advances in high‐throughput experimental enzymology, such as HT‐MEK (Markin et al., 2021; Muir et al., 2025), have begun generating large‐scale, sequence‐resolved kinetic datasets. These efforts highlight the growing need for structured, interoperable datasets that link enzyme sequence, substrate, and kinetic parameters across both legacy literature and new experimental sources. Bridging this gap calls for automated pipelines capable of formatting, standardizing, and aligning extracted data with existing protein and compound databases.

To address these limitations, we developed EnzyExtract, an AI‐powered pipeline designed specifically to extract, validate, and structure quantitative enzyme kinetic data from the scientific literature at scale. EnzyExtract goes beyond existing approaches by targeting not only the enzyme‐substrate relationships but also the specific kinetic parameters (*k*_cat_, *K*_*m*_) and experimental conditions (pH, temperature, mutations) that are essential for predictive modeling of enzyme functions and properties (Jurich et al., 2025). By leveraging a fine‐tuned version of GPT‐4o‐mini, specialized optical character recognition, and sophisticated entity disambiguation techniques, EnzyExtract efficiently processes tens of thousands of full‐text publications to create a comprehensive, sequence‐mapped enzymology database. We have successfully extracted 218,095 enzyme‐substrate‐kinetic data points entries from 137,892 scientific publications. These datapoints were further curated into 176,463 enzyme–substrate–sequence entries categorized by data confidence: 92,286 high‐confidence entries, 54,550 medium‐confidence entries, and 29,627 low‐confidence entries. This curated dataset has been organized into a structured database, EnzyExtractDB. Each data point is enriched with critical contextual information and aligned with established reference databases such as UniProt (Consortium, U, 2015) and PubChem (Kim et al., 2023), enabling immediate integration with computational workflows. Rigorous validation against manually curated datasets demonstrates the accuracy of our extraction process, and retraining of state‐of‐the‐art enzyme kinetics predictors with our data shows significant improvements in predictive performance. EnzyExtract represents an advance in automated enzyme data extraction. By illuminating the “dark matter” of enzymology, EnzyExtract provides a valuable resource for the scientific community.

## 2 METHOD AND IMPLEMENTATION

### 2.1 Development of the EnzyExtract pipeline

**FIGURE 1:**
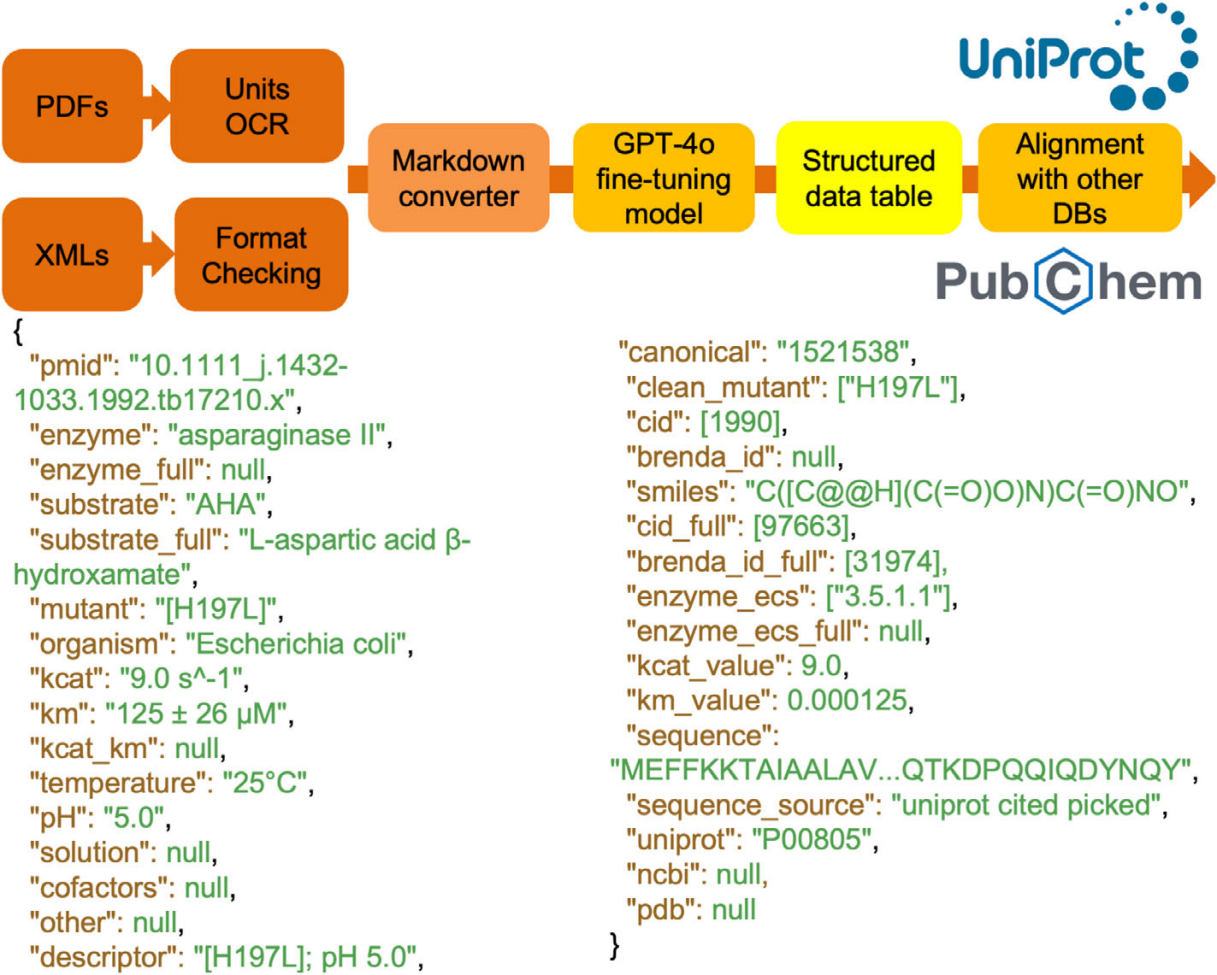
Overview of the enzymatic kinetics data extraction and processing pipeline. The first step involves retrieving 137,892 scientific papers from the OpenAlex API using a set of curated keywords derived from BRENDA and SABIO‐RK (e.g., “*k*_cat_”, “kinetics”). The retrieved articles in PDF and XML formats are then parsed using the PyMuPDF library, and an optical character recognition (OCR) model (ResNet18) is employed to extract figures and tables. To enhance unit recognition, a fine‐tuned OCR model is applied to standardize numerical values and units within extracted tables. Once the data is converted into plain text, a fine‐tuned GPT‐4o model processes the extracted information to generate structured JSON files containing enzymatic reaction conditions, including enzyme name, organism, substrate name, pH, temperature, *k*_cat_, *K*_*m*_, and other reaction parameters.

The EnzyExtract pipeline begins with the large‐scale acquisition of full‐text scientific literature, sourced from multiple publishing platforms and enriched with enzymology‐specific content (Figure 1). To construct this ensemble, we queried OpenAlex using 13 targeted keywords related to *k*_cat_ and Michaelis–Menten kinetics, retrieving 60,567 PDF and 85,534 XML files (146,101 publications in total). A complementary search on Web of Science with 16 additional terms further expanded the dataset (see Text S1 for the full list of keywords). Document identifiers, including DOIs and PubMed IDs, were collected via indexing services, and full‐text access was obtained through Elsevier and Wiley's text and data mining APIs, while open‐access documents were retrieved using the OpenAlex REST API (Priem et al., 2022). After excluding documents with duplication, entry errors, or abstract‐only content, the final ensemble was refined to 137,892 unique articles. These documents, spanning both PDF and XML formats, were parsed using custom scripts that segment textual content, figures, and tables. A ResNet‐18 model (He et al., 2016), fine‐tuned with two fully connected layers, was employed to correct recognition of kinetic parameter units during PDF parsing. This allows EnzyExtract to recover enzymology‐relevant data across diverse publication formats, including information often overlooked by traditional text‐only pipelines.

Following the initial extraction of raw content for XML files (using BeautifulSoup; Richardson, 2007) and PDFs (using PyMuPDF; Adhikari & Agarwal, 2024), a standardization pipeline is applied to harmonize formatting and prepare the data for downstream analysis (Figure 1). Tables embedded within PDF documents are processed using the TableTransformer model (Smock et al., 2022), and are subsequently converted into Markdown format to ensure structural consistency during parsing. To assess parsing fidelity across data sources, we benchmarked two Markdown conversion strategies, OCR‐based parsing and PyMuPDF‐based extraction, on a shared test set of 384 enzyme‐related documents (Table S1). The results show notable differences in structural accuracy, runtime efficiency, and downstream parse success rates. Specifically, incorporating TableTransformer as a deep‐learning‐based table preprocessing step enhances alignment between table values and headers, thereby improving structural accuracy. This is reflected in an increase in precision from 0.71 to 0.80 and recall from 0.68 to 0.86, resulting in a higher F1‐score (0.69 vs. 0.83) and accuracy (0.53 vs. 0.71) on *k*_cat_ extraction (see Table S1a). In contrast, a ResNet‐18‐based preprocessing step had minimal effect on *k*_cat_ extraction but improved *K*_*m*_ accuracy due to better unit classification, which improves accuracy from 0.46 to 0.66 on *K*_*m*_ extraction (Table S1b). However, TableTransformer comes with a trade‐off in runtime efficiency: it can process ~500 PDFs in 15 min, whereas PyMuPDF processes them in just 30 s. Nonetheless, the improvements in structural fidelity and downstream parse success rates justify the additional computational cost. As such, we adopted OCR preprocessing combined with TableTransformer‐based table extraction in our final pipeline to maximize overall performance in enzyme kinetic data extraction.

The curated textual and tabular data are then passed through a fine‐tuned large language model (GPT‐4o‐mini‐2024‐07‐18), trained on 62 manually curated articles (52 for training, 10 for validation) over three epochs with a batch size of 3 and a learning rate of 1.8 (SI.zip). This model is optimized to identify and extract essential enzymology metadata, including enzyme names, organism source, substrate identity, pH, temperature, as well as kinetic parameters (*k*_cat_ and *K*_*m*_), encoding them into structured JSON entries (*bottom*, Figure 1). Compared to the base GPT‐4o model, the fine‐tuned EnzyExtract pipeline improves metadata extraction accuracy—particularly in row name identification, and delivers robust, balanced performance across both *k*_cat_ (F1 = 0.83) and *K*_*m*_ (F1 = 0.80) extractions. While Claude 3.7 achieves slightly higher precision on some tasks, EnzyExtract matches or exceeds it in recall, all while operating at just 1/30th the inference cost, making it a more scalable and cost‐effective solution for high‐throughput enzyme kinetics extraction (see Table S1). This evaluation was performed on another manually curated set of 384 literature documents used in the OCR benchmarking (SI.zip). At this stage of the pipeline, a total of 242,116 raw data entries have been extracted from the processed PDFs and XMLs. After removing duplicate rows, excluding documents present in both PDF and XML forms, and excluding all entirely empty rows lacking any data in the enzyme, substrate, or kinetics columns, we obtained a total of 218,095 rows, including 218,095 *k*_cat_ and 167,794 *K*_*m*_ values.

To enhance data accuracy, we systematically examined and marked the data. We detected LLM hallucinations by checking the original PDF or XML document for the reported numeric values. If a significant proportion of numeric values cannot be located by regex search, then we consider the LLM response to be hallucinated. Likewise, LLMs are known to generate repetitive content (Yang et al., 2023). Papers with an insufficient proportion of unique numerical values are excluded. Finally, rows with scientific notation are marked as possibly inaccurate due to ambiguity in the sign of the exponent. 15,444 rows (7.1%) are marked as possibly hallucinated; 9630 (4.4%) as repetitive; and 20,890 (9.6%) as containing scientific notation. Though numerous legitimate values are present in these rows, we exclude them to enhance the overall quality of the dataset.

After these curation steps, 176,463 *k*_cat_ and 135,527 *K*_*m*_ values remain. This dataset was compiled into a new database named EnzyExtractDB (see Data and Software Availability section). The distribution of reported assay temperatures spans from 0°C to 100°C, with an average of 25.9°C, while pH values range from 2.0 to 12.0, averaging 7.2 (Figure S1). The database encompasses 3569 unique four‐digit EC numbers. Based on the distribution of the first digit of EC numbers, a total of 84,464 entries are classified, comprising EC 1 (oxidoreductases): 27,879; EC 2 (transferases): 16,301; EC 3 (hydrolases): 27,514; EC 4 (lyases): 5507; EC 5 (isomerases): 3128; EC 6 (ligases): 3044; and EC 7 (translocases): 1091 (Figure S1). Log_10_‐transformed kinetic parameters exhibit broad ranges as well, with Log_10_(*k*_cat_) values from −5.8 to 5.3 and Log_10_(*K*_*m*_) values from −5.5 to 4.5, both displaying approximately Gaussian distributions (Figure S2).

To integrate with existing databases, we cross‐referenced the metadata: enzyme names are linked to UniProt accession numbers, and substrate names are translated into SMILES strings via PubChem. For the substrate names represented as abbreviations, EnzyExtract employs GPT‐4o to recover the full chemical names prior to querying PubChem. In cases where multiple PubChem matches are returned, the top‐ranked result is selected. A key challenge arises from the frequent omission of explicit sequence identifiers in the source literature. To address this, EnzyExtract retrieves up to five metadata fields from each article, including: enzyme name, organism name, UniProt ID, NCBI accession number, and PDB ID. If any of the identifiers (e.g., UniProt ID or NCBI accession) are available, they are used to directly retrieve the corresponding sequence. If no identifier is present, a combined search using enzyme and organism names is performed against UniProt, and the top‐ranked hit is selected. To evaluate the fidelity of sequence retrieval in a quantitative manner, we implemented a confidence scoring system based on the number of metadata fields successfully extracted from each article. Entries were classified as “high‐confidence” if at least three out of five metadata fields were retrieved, “medium‐confidence” if two fields were present, and “low‐confidence” if only a single field could be extracted. By automating what was once a manual and error‐prone curation process, EnzyExtract not only scales data collection but also ensures compatibility with existing enzymology database data structures. As a result, we curated a total of 176,463 enzyme–sequence–substrate pairs, comprising 92,286 high‐confidence entries, 54,550 medium‐confidence entries, and 29,627 low‐confidence entries.

### 2.2 Enhancement of the data extraction fidelity

One of the most deceptively simple yet impactful challenges in enzymology data extraction lies in correctly interpreting measurement units, especially distinguishing between the Greek letter “μ” (micro) and the lowercase “m” (milli). This subtle difference can lead to a thousand‐fold errors in kinetic constants like *k*_cat_ and *K*_*m*_, compromising the biological validity of the extracted data. Automated optical character recognition (OCR) tools frequently misclassify these symbols, especially in low‐resolution figures and scanned PDFs, posing a critical barrier to accurate downstream data structuring.

**FIGURE 2:**
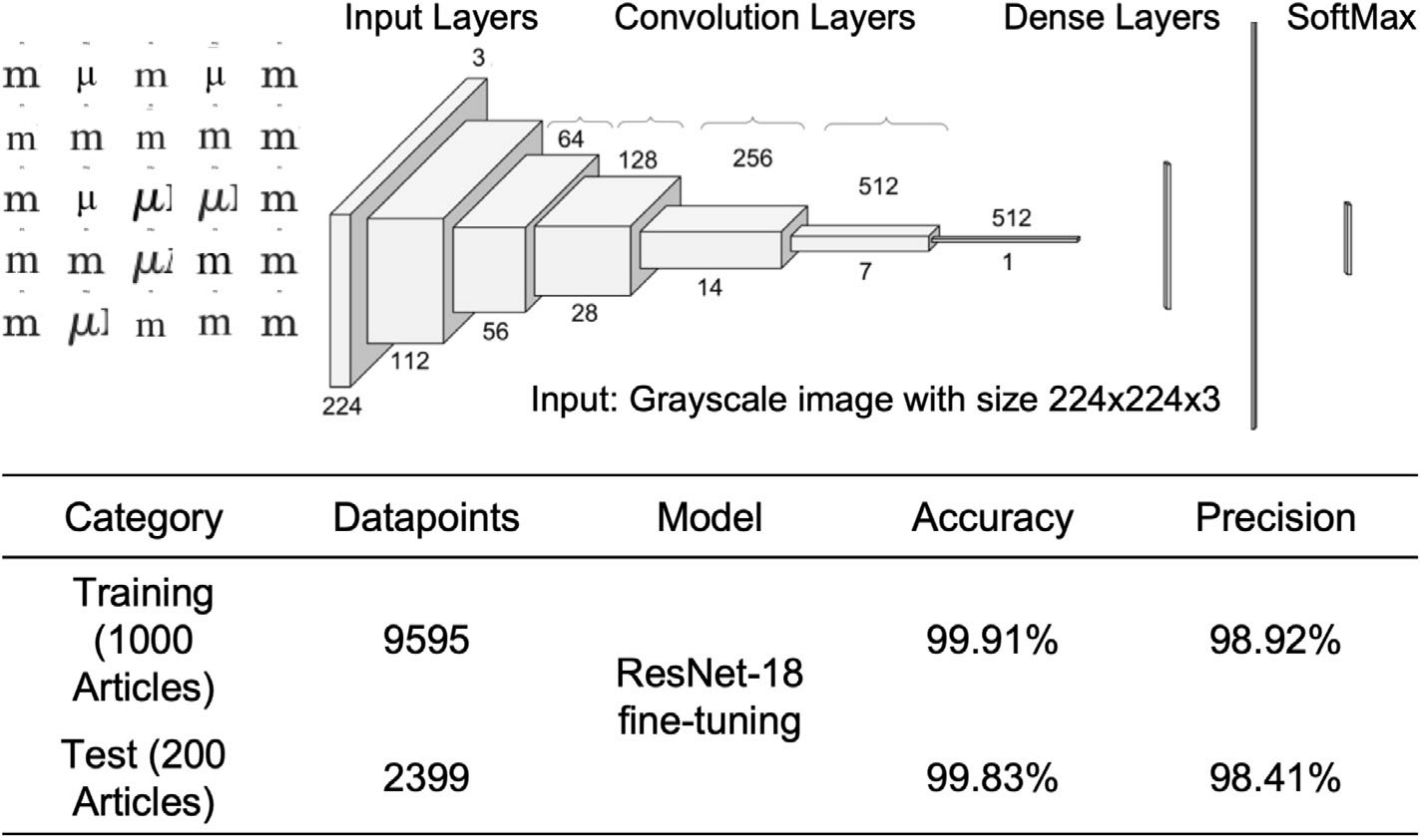
Overview of the optical character recognition (OCR) pipeline. Accurate recognition of the “μ” (micro) symbol is essential, as confusion with the “m” (milli) symbol can lead to errors by a factor of 1000. To address this, we curated a dataset of 11,994 labeled symbol instances, comprising 5295 instances of “m”, 6103 instances of “μ”, and 596 instances of other symbols. From this dataset, symbols were extracted from 1200 scientific articles: 1000 articles were used for training (9595 symbols), and 200 were held out for testing (2399 symbols). AResNet‐18 model pretrained on ImageNet‐1k (He et al., 2016) was fine‐tuned using transfer learning, achieving 99.8% accuracy on the test set after 10 epochs. Figures and tables were parsed using PyMuPDF, and the fine‐tuned ResNet‐18 model was applied to ensure accurate symbol recognition. The recognized content was then converted into structured JSON using a fine‐tuned GPT‐4o model, capturing key experimental details such as enzyme name, organism, substrate, pH, temperature, *k*_cat_, *K*_*m*_, and other reaction parameters for downstream analysis and integration into a searchable database.

To address unit misrecognition issues, particularly distinguishing between visually similar glyphs like “m” and “μ”, we developed a custom unit recognition module by fine‐tuning a ResNet‐18 convolutional neural network (He et al., 2016) on a curated dataset of 11,994 symbol instances sourced from 1200 scientific articles (Figure 2). This dataset includes a balanced mix of 5295 instances of “m” 6103 instances of “μ” and 596 examples of other confusable characters. The model was trained for 10 epochs using the Adam optimizer with a learning rate of 0.001 on 9595 training samples and evaluated on an independent test set of 2399 symbols. It achieves an accuracy of 99.8% on the test set and 99.5% on an extended benchmark of 17,433 images, demonstrating strong generalization across diverse formatting styles, font resolutions, and document layouts (Figure 2).

Accurate unit recognition is essential for preserving the integrity of kinetic values during the GPT‐4o‐based extraction process. The resulting clean data was then passed to the GPT‐4o model, which could associate reaction parameters with the correct quantitative context, enabling precise and reliable structuring of enzymology datasets at scale.

## 3 RESULTS AND DISCUSSION

### 3.1 Validation of the EnzyExtract pipeline

To ensure the reliability of enzymatic kinetic parameters extracted by EnzyExtract, we benchmarked its data extraction performance against both a manually curated gold‐standard dataset and the widely used BRENDA database (Table 1). The ground‐truth dataset was constructed by re‐curating 223 scientific articles previously indexed by BRENDA (see SI.zip). Each entry in this dataset was carefully annotated to include a unique key—composed of enzyme name, mutation identifier (if applicable), substrate name, and organism name—and its associated value, namely *k*_cat_ or *K*_*m*_ along with their corresponding units. This standardized key–value pair allows for consistent comparison across datasets. EnzyExtract was evaluated by scanning the same literature ensemble to generate comparable key–value pairs, enabling a parallel and fair assessment relative to BRENDA.

To systematically assess extraction quality, we employed strict alignment criteria. An optimal pairing was found with linear sum assignment (Schilling‐Wilhelmi et al., 2025), and enzyme names and organism descriptors were considered a match if they achieved at least 90% string similarity, while substrate alignment required either an exact string match or identical SMILES representation. Only upon successful alignment of both enzyme and substrate descriptors were the numerical values and units of *k*_cat_ or *K*_*m*_ compared (see Text S3). Under these criteria, a true positive (TP) is defined as a case where the extracted entry matched the ground truth in both key and numerical value (including correct units). A false negative (FN) occurred when the model failed to extract any value for a valid key present in the ground truth. A false positive (FP) referred to cases where a partially correct extraction was made—e.g., a correct key but an incorrect or mismatched value. Cases where no matching key was expected and none was returned were considered true negatives (TN), although these cannot be explicitly reported due to the nature of the benchmarking design (Schilling‐Wilhelmi et al., 2025). Evaluating precision is particularly critical in this context because the errors in these numerical values can propagate through downstream analyses and affect the fidelity of predictive enzyme models for systems biology and enzyme engineering (Schilling‐Wilhelmi et al., 2025). We also reported a distinct category termed “Unresolved” which denotes entries for which neither a correct enzyme–substrate pair nor a corresponding numerical value could be identified. These cases represent complete mismatches and are not included in the calculation of accuracy or precision, as they fall outside the defined true positive, false positive, and false negative categories.

Using this framework, EnzyExtract achieves an accuracy of 0.80 and a precision of 0.84 for *k*_cat_ values, and an accuracy of 0.76 and a precision of 0.79 for *K*_*m*_ (Table 1a). Twenty *k*_cat_ values are off by a factor of 10^3^, which is the result of misrecognized scientific notation from one PMID. For *K*_*m*_, 49 values are off by 1000‐fold, likely due to unit misrecognition or glyph confusion such as “μ” versus “m”. In contrast, BRENDA's curated entries for the same literature exhibit comparable accuracy (0.67 for *k*_cat_, 0.78 for *K*_*m*_) and precision (0.83 for *k*_cat_, 0.78 for *K*_*m*_), indicating significant challenges in consistency even within user‐curated resources (Table 1b).

**TABLE 1:** Data validation results for EnzyExtract (a) and BRENDA (b), using manually curated datasets as ground truth.

EnzyExtract vs. ground truth data	FP	FN	TP	Off by 10	Off by 10^2^	Off by 10^3^	Accuracy	Unresolved	Precision
*k*_cat_	59	17	301	0	0	20	0.80	779	0.84
*K*_*m*_	127	26	486	3	1	49	0.76	560	0.79
BRENDA vs. ground truth data	FP	FN	TP	Off by 10	Off by 10^2^	Off by 10^3^	Accuracy	Unresolved	Precision
*k*_cat_	16	23	80	0	0	0	0.67	2489	0.83
*K*_*m*_	314	9	1134	18	0	51	0.78	1450	0.78

*Note*: The alignment process consists of three steps: (1) matching enzyme name and organisms with a 90% similarity cutoff, (2) aligning substrate structures using 100% similarity on SMILES strings, and (3) matching kinetic parameters (*k*_cat_, *K*_*m*_) based on the previous two steps. False Positives (FP), False Negatives (FN), and True Positives (TP) follow standard statistical definitions, using *k*_cat_ as the reference metric. Accuracy is defined as $\frac{\mathrm{TP}}{\mathrm{TP}+\mathrm{FP}+\mathrm{FN}}$ and Precision as $\frac{\mathrm{TP}}{\mathrm{TP}+\mathrm{FP}}$. “Off by 10”, “Off by10^2^”, and “Off by 10^3^” refer to extracted values that differ from the ground truth by corresponding numerical factors. “Unresolved” indicates cases where no match was found in the first two alignment steps.

These results highlight EnzyExtract's robustness and accuracy for automated data extraction at scale. Compared to BRENDA, EnzyExtract exhibits comparable or slightly superior performance (for *k*_cat_) across all metrics, with fewer large‐scale numerical discrepancies and a stronger ability to correctly associate quantitative values with biochemical contexts. This positions EnzyExtract as a trustworthy alternative for kinetic data curation, capable of supporting large‐scale enzymology initiatives with both accuracy and efficiency.

**FIGURE 3:**
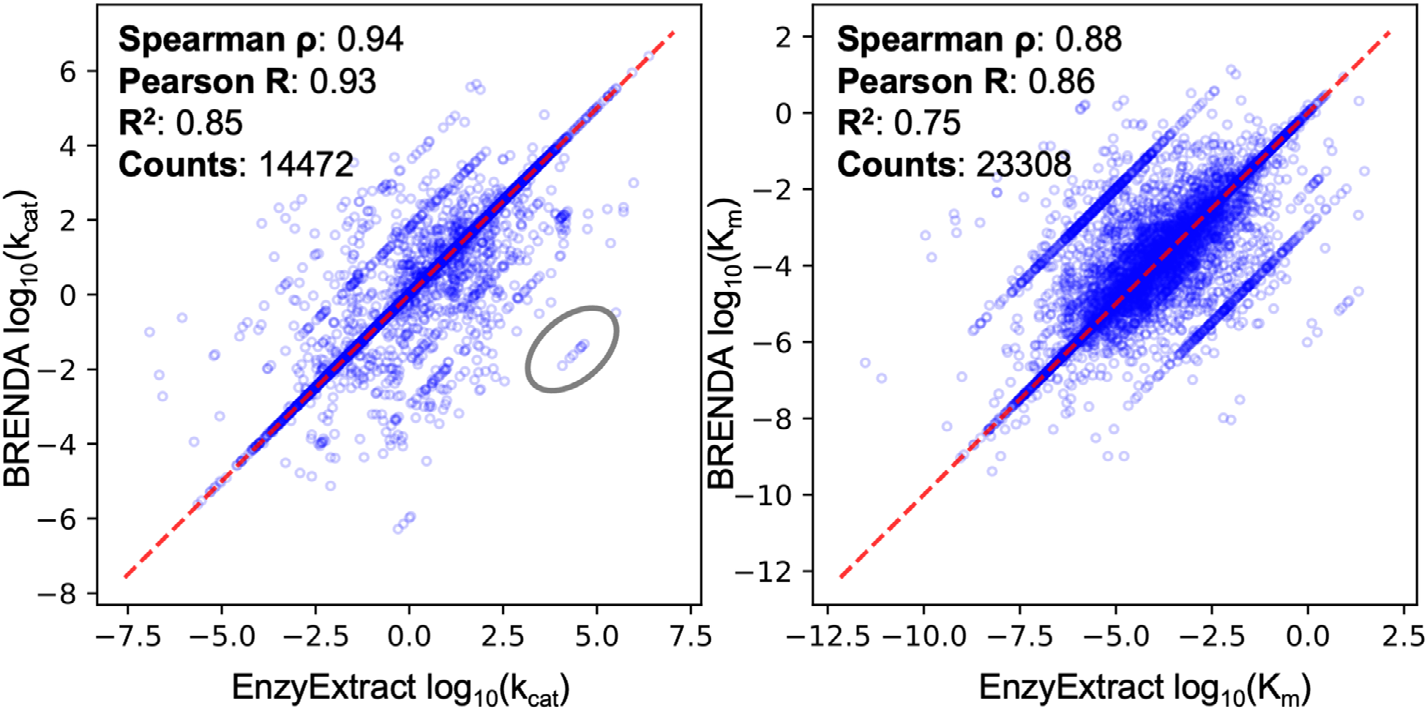
Correlation analysis of kinetic parameters extracted by EnzyExtract and those from the BRENDA database. The left plot compares log_10_(*k*_cat_) values, while the right plot compares log_10_(*K*_*m*_) values. Each point represents a matched enzyme‐substrate pair, with EnzyExtract‐extracted values plotted against their corresponding BRENDA values. Strong correlations are observed, with Spearman ρ = 0.94 and Pearson *R* = 0.93 for *k*_*cat*_ (*R* ^2^ = 0.85), and Spearman's ρ = 0.88 and Pearson's *R* = 0.86 for *K*_*m*_ (*R* ^2^ = 0.75). These results show the reliability of EnzyExtract's pipeline for extracting kinetic constants and demonstrate its potential utility in identifying systematic inconsistencies present in manually curated databases. Rather than replacing human curation, EnzyExtract provides a scalable, interpretable layer of validation that enhances the fidelity of biochemical databases, particularly in contexts where unit precision plays a critical role in downstream modeling and inference.

To further evaluate the consistency of the extracted kinetic parameters at scale, we conducted a correlation analysis between values extracted by EnzyExtract and those reported in the BRENDA database. We aligned 14,472 *k*_cat_ and 23,308 *K*_*m*_ datapoints by matching identical enzyme–substrate pairs, applying the same criteria as in our earlier benchmark: ≥ 90% name similarity for enzymes and a 100% exact match for substrate names or SMILES strings. This large‐scale comparison reveals strong statistical consistency between EnzyExtract and BRENDA across both parameters (Figure 3). For *k*_cat_, Spearman's ρ was 0.94 and Pearson's *R* was 0.93 (*R* ^2^ = 0.85), with a mean absolute deviation (MAD) of 0.14 and root mean square deviation (RMSD) of 0.57. For *K*_*m*_, Spearman's ρ reached 0.88 and Pearson's *R* was 0.86 (*R* ^2^ = 0.75), with a MAD of 0.19 and RMSD of 0.70. These low deviation values indicate that most EnzyExtract‐derived values fall within a narrow margin of their BRENDA‐reported counterparts, even when extracted from unstructured full texts. Notably, the slightly greater deviation values observed for *K*_*m*_ may stem from the greater contextual ambiguity in units and conditions reported for Michaelis constants, as well as the broader dynamic range of valid *K*_*m*_ values. Nonetheless, these metrics confirm that EnzyExtract yields kinetic data quantitatively consistent with manually curated data, validating its use for large‐scale, high‐throughput extraction.

Visual inspection of the scatter plots offers additional insight. While the majority of datapoints cluster along the identity line (y = x), several secondary clusters appear in parallel trajectories, with one cluster highlighted in a gray circle in Figure 3, suggesting consistent value shifts by fixed logarithmic intervals. These patterns are likely indicative of unit inconsistencies in BRENDA, such as misinterpretations between s^−1^ and min^−1^ for *k*_cat_, or between μM and mM for *K*_*m*_. Notably, such discrepancies are more likely to arise from legacy literature or earlier curation pipelines where unit markings, especially those involving visually similar glyphs such as “μ” and “m”, may be difficult to discern due to font resolution, scanning artifacts, or PDF degradation. Notably, EnzyExtract incorporates a unit‐aware recognition module specifically designed to resolve ambiguities in visual typography. By integrating convolutional neural network models trained to disambiguate confusable units, the system is better positioned to flag and correct systematic errors that might be introduced during manual annotation. In contrast, manual curation efforts, while meticulous, may not consistently detect such errors, particularly when reviewing older or low‐quality sources.

Further examination of the outliers (highlighted with a gray circle) identified six data points with substantial deviations attributable to apparent inaccuracies in BRENDA. Manual reinspection of the original sources (PubMed IDs: 14561748, 25577493, 12021281, 15010546, 15200387, and 15746370) confirms that in these cases EnzyExtract's extracted values accurately reflect the published figures, whereas discrepancies in BRENDA are due to misassigned units or numerical misreadings during prior curation.

**FIGURE 4:**
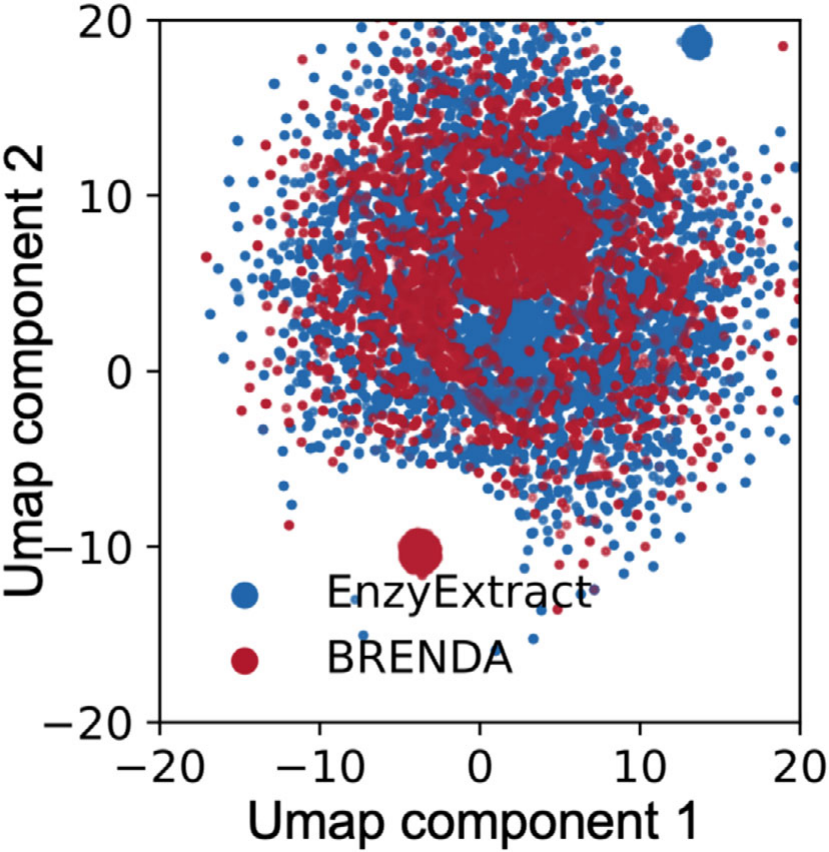
UMAP visualization of enzymology kinetics datasets comparing EnzyExtract (blue) and BRENDA (red). Key features used for embedding include organism, enzyme name, substrate, and mutations, which were label‐encoded before applying UMAP with n_neighbors = 15 and min_dist = 0.1 for dimensionality reduction. The visualization reveals that EnzyExtract provides broader coverage, capturing additional enzyme‐substrate relationships beyond those in BRENDA. The distributional differences suggest that EnzyExtract can uncover new enzymology knowledge from unstructured sources, complementing existing databases.

EnzyExtract's final raw output includes 218,095 datapoints, surpassing BRENDA's 157,000 raw entries prior to sequence or SMILES filtering. For key biological entities, EnzyExtract consistently involves higher diversity: 22,687 unique enzyme names versus 4821 in BRENDA, 6245 unique organisms compared to BRENDA's 3732, and 16,070 unique mutation types versus 15,724 in BRENDA. To assess the breadth of enzyme–substrate pairs captured by EnzyExtract, we conducted a dimensionality reduction analysis using UMAP on the combined datasets from EnzyExtract and BRENDA. Key categorical features, including enzyme name, organism, substrate identity, and site‐specific mutations, were label‐encoded and projected into a 2D space using UMAP (n_neighbors = 15, min_dist = 0.1, Figure 4). The resulting visualization reveals a broader distribution for EnzyExtract (blue) compared to BRENDA (red), with EnzyExtract's entries forming a denser and more expansive coverage of the enzymology space. In contrast, BRENDA exhibits tighter clustering, reflecting its reliance on historical accumulation, selective manual curation, and an emphasis on canonical enzymatic pathways. EnzyExtract's broader coverage suggests its capacity to extract underrepresented or emerging enzyme mutant–substrate pairs from unstructured literature sources, which is evidenced by a more extensive region in the mutational embedding space (Figure S4).

Notably, EnzyExtract yields fewer distinct substrate entries: 8294 versus BRENDA's 12,000. We attribute this gap to two main factors. First, the LLM backbone of EnzyExtract may struggle with generic or ambiguous polymeric substrate names (e.g., DNA, RNA, or proteins), which often appear without accompanying structural descriptors. Second, the current extraction pipeline lacks an image‐to‐SMILES module, limiting its ability to interpret molecular structures embedded as figures or diagrams in PDF files (Rajan et al., 2023). These constraints are particularly evident in instances where substrate identity is provided only as images in the original literature, limiting automated extraction and downstream analyses. Despite these limitations, the substrate space represented in EnzyExtractDB spans a broad range of chemical diversity (see Figure S3 for Morgan fingerprint‐based clustering).

### 3.2 Application: Enhancing predictive modeling for enzyme kinetics

To evaluate the impact of EnzyExtract‐derived large‐scale dataset on predictive modeling of a total 44,376 datapoints, we retrained three previously published machine learning kcal predictors (i.e., MESI [Nie et al., 2024], DLKcat [Li et al., 2022], and TurNuP [Kroll et al., 2023]) using a curated training set of 36,980 kcat entries extracted via EnzyExtract. Performance was assessed on a held‐out test set of 7396 entries, evaluated using five performance metrics: root mean squared error (RMSE), mean absolute error (MAE), Pearson's correlation coefficient (*R*), coefficient of determination (*R* ^2^), and Spearman's rank correlation coefficient (ρ) (Table 2). These three models differ in both architectural design and dataset splitting strategy. DLKcat (Li et al., 2022), a sequence‐based neural network model, uses a random split of enzyme–substrate pairs without controlling for sequence similarity, causing identical or highly similar enzymes to appear in both training and test sets. TurNuP (Kroll et al., 2023) incorporates protein language models and attention mechanisms, splitting data by enzyme so that each unique enzyme sequence appears in only one dataset partition, with test performance further stratified by sequence identity to the training set. MESI (Nie et al., 2024) adopts a similar approach, assigning enzymes to distinct splits and evaluating performance across defined sequence identity thresholds, including on remote homologs with <40% similarity. In our retraining experiments, we followed each model's original data‐splitting protocol to ensure consistency and comparability of performance (see Text S4 for details).

**TABLE 2:** Performance comparison of kinetic parameter prediction models retrained using EnzyExtract‐derived data.

Models	RMSE (s^−1^)	MAE (s^−1^)	Pearson R	*R* ^2^	Spearman ρ
MESI	1.42	1.36	0.87	0.65	0.82
MESI (original)	1.86	1.94	0.72	0.46	0.69
DLKcat	1.54	1.32	0.80	0.59	0.79
DLKcat (original)	2.31	1.98	0.67	0.43	0.64
TurNuP	1.73	1.23	0.83	0.62	0.86
TurNuP (original)	1.79	1.74	0.74	0.57	0.72

*Note*: The models were trained on 36,980 data points and evaluated on 7396 test data points, with key performance metrics reported, including RMSE, MAE, Pearson *R*, *R* ^2^, and Spearman ρ. Retrained models (MESI, DLKcat, and TurNuP) using EnzyExtract data show significant performance improvements over their original versions, with reductions in RMSE and MAE, and increases in Pearson's *R*, *R* ^2^, and Spearman's ρ. These results demonstrate the benefit of incorporating EnzyExtract‐derived enzymology data for enhancing predictive accuracy in kinetic parameter estimation.

Across all models, retraining with EnzyExtract data led to improvements in predictive performance. MESI achieves an RMSE of 1.42 s^−1^ and MAE of 1.36 s^−1^—marked enhancements over its original implementation (RMSE 1.86 s^−1^, MAE 1.94 s^−1^)—as well as higher Pearson *R* (0.87 vs. 0.72), *R* ^2^ (0.65 vs. 0.46), and Spearman ρ (0.82 vs. 0.69). DLKcat also exhibits improved predictive accuracy following retraining (RMSE 1.54 s^−1^, *R* ^2^ 0.59) relative to its original benchmark (RMSE 2.31 s^−1^, *R* ^2^ 0.43). TurNuP, despite being a strong baseline model, also benefits from the enriched dataset and achieves the lowest MAE (1.23 s^−1^ vs. original: 1.74 s^−1^) and the highest Spearman's ρ (0.86 vs. original: 0.72) among the three.

These findings underscore the utility of larger‐scale enzymology datasets for developing robust predictive models. By expanding the volume and diversity of training data beyond manually curated repositories, EnzyExtract enables improved model generalization across a broader enzymatic landscape. Consequently, future modeling efforts leveraging such data are likely to achieve greater accuracy, robustness, and practical relevance for enzyme design, metabolic modeling, and biotechnological innovation.

## 4 DISCUSSION

In this study, we presented EnzyExtract, a high‐throughput and fully automated pipeline for extracting structured enzymology data from full‐text scientific literature using large language models. By integrating OCR, table parsing, metadata resolution, and prompt‐engineered GPT‐4 extraction, EnzyExtract enables the systematic recovery of relational data linking enzyme sequence, substrate identity, kinetic parameters, and assay conditions. The pipeline extracted over 242,116 raw enzyme‐substrate‐kinetic datapoints, which were further curated into 176,463 enzyme–substrate–sequence entries categorized by data confidence: 92,286 high‐confidence entries, 54,550 medium‐confidence entries, and 29,627 low‐confidence entries. This stratification allows researchers to balance data quantity with quality in downstream applications. To demonstrate its utility, we retrained state‐of‐the‐art kinetic predictors—including DLKcat, MESI, and TurNuP—using EnzyExtract‐derived datasets. All models showed consistent improvements in RMSE, MAE, and *R* ^2^ on held‐out test sets, confirming that high‐confidence, literature‐mined data can meaningfully enhance predictive enzyme modeling.

While EnzyExtract demonstrates strong performance in extracting enzyme kinetic parameters at scale, several limitations remain that present opportunities for future improvement. First, our current implementation relies solely on GPT‐4 for language understanding and extraction. We did not benchmark or compare performance across other large language models (LLMs) such as Claude, Gemini, or open‐source models like Mistral or DeepSeek, which may offer complementary strengths or cost efficiencies. Evaluating a broader set of LLMs, as demonstrated in the Co‐Scientist (Jiang et al., 2025) study, could boost EnzyExtract's robustness and generalizability.

Second, while the current pipeline operates in a fully automated manner, expert‐level accuracy still necessitates manual curation. At present, the system incorporates only minimal safeguards against errors, relying primarily on heuristic filters to identify hallucinations and repetitive outputs. To enhance reliability, future iterations could adopt a hybrid framework in which algorithmic predictions are systematically reviewed, corrected, or prioritized by human annotators—particularly in cases involving ambiguity or low‐confidence extractions. Such a collaborative approach, integrating automated scalability with expert oversight, has the potential to substantially improve overall accuracy.

Third, EnzyExtract currently concentrates on extracting kinetic parameters such as *k*_cat_ and *K*_*m*_ while assay conditions and reaction details are only partially captured. The present pipeline can retrieve a limited set of experimental conditions, including assay pH, temperature, and cofactors; however, many additional functional descriptors frequently reported in the literature—such as enzyme stability (e.g., melting temperature, half‐life), optimal temperature profiles, product identities and yields, reaction stoichiometry, inhibition constants (*K*_*i*_) and mutational effects—remain unexploited (Xie & Warshel, 2023). Expanding extraction capabilities to include these parameters would significantly enrich enzyme knowledge bases, support more detailed mechanistic studies, and improve the development of predictive models for enzyme design and engineering. As the extraction scope broadens, downstream data curation will require context‐specific filtering; for instance: selecting datasets with complete kinetic and product annotations for pathway modeling or isolating high‐confidence mutational and inhibition data for machine learning‐based activity prediction.

Despite its ability to extract large volumes of kinetic data with structured metadata, the current version of EnzyExtract lacks experiment‐level standardization, particularly in distinguishing or aggregating identical enzyme–substrate pairs measured under varying assay conditions across different laboratories. At present, the pipeline does not group kinetic measurements based on experimental protocols, nor does it incorporate controlled vocabularies or ontological frameworks to assess the reproducibility or comparability of reported values. Instead, data harmonization is performed statistically, based on extracted numerical features and confidence scores, without formally accounting for contextual experimental variability. Although sequence and substrate identifiers can often be matched to external resources such as UniProt and PubChem, the fraction of entries for which complete metadata—such as pH, temperature, or cofactor information—can be reliably linked remains limited. To improve the granularity, quality, and reusability of extracted datasets, future versions of EnzyExtract could adopt formal reporting standards such as EnzymeML (Lauterbach et al., 2023) and STRENDA (Swainston et al., 2018). EnzymeML provides a machine‐readable markup format for storing kinetic measurements along with detailed reaction conditions, while STRENDA Guidelines define minimum information requirements for reporting enzymology data. Integration with STRENDA DB, which is a curated, DOI‐registered repository for compliant kinetic datasets, would facilitate rigorous validation of extracted parameters and promote reproducibility across publications. By aligning with these standards, EnzyExtract could better support benchmarking, comparison, and reuse of enzymology data across studies, ultimately advancing its utility for machine learning applications and mechanistic modeling.

While the identifier‐based resolution strategy employed by EnzyExtract significantly enhances the ability to retrieve enzyme sequences and substrate structures, it does not guarantee high fidelity to the exact molecular entities used in the original kinetic experiments. In many cases, the specific protein construct employed in an enzymology study, such as a mutant, fusion tag, or truncated variant, is not explicitly reported in the manuscript. Instead, researchers may reference a canonical template sequence through a UniProt or PDB ID without noting experimental modifications, leading to discrepancies when aligning extracted metadata to external databases. As a result, the inferred sequences may represent the closest available approximation, but not necessarily the precise variant tested in the assay.

A similar limitation applies to substrate annotation: while common small molecules can typically be mapped to PubChem using name‐based searches, complex substrates such as protease targets, nucleic acids (e.g., DNA, RNA), and polysaccharides (e.g., starch) often lack clear SMILES string representations or are ambiguously named in the literature. In such cases, the current pipeline is unable to reliably resolve the substrate to a canonical chemical structure, highlighting an area where future model improvements, such as the integration of domain‐specific ontologies or experimental metadata standards, could further enhance the precision and completeness of the extracted dataset. In parallel, product information, such as product structure, yield, and specificity (Xing et al., 2024), is inconsistently reported and currently not captured by EnzyExtract. Including such data would provide valuable mechanistic context and support a wider range of applications, including pathway modeling, yield optimization, and selectivity prediction. Together, improved resolution of both substrate and product entities would directly benefit downstream tools such as EnzyKR (Ran et al., 2023), CLEAN (Yu et al., 2023), which rely on accurate molecular representations to predict enzyme–substrate selectivity and reaction outcomes.

Finally, integration with computational enzymology platforms such as EnzyHTP (Shao et al., 2022, 2023, 2025), AlphaFold2 (Jumper et al., 2021), or Rosetta (Ahern et al., 2025) could streamline structure–function pipelines and enable closed‐loop design and discovery. By embedding EnzyExtract as a modular data layer, the broader vision of a fully automated enzyme engineering and annotation platform comes closer to reality.

## AUTHOR CONTRIBUTIONS

**Galen Wei:** Methodology; software; data curation; investigation; writing – original draft; writing – review and editing. **Xinchun Ran:** Conceptualization; investigation; methodology; software; data curation; writing – original draft; writing – review and editing. **Runeem AI‐Abssi:** Methodology; investigation; validation; data curation. **Zhongyue Yang:** Conceptualization; writing – original draft; writing – review and editing; project administration; resources; methodology; investigation; funding acquisition.

## CONFLICT OF INTEREST STATEMENT

The authors declare no competing financial interest.

## Supporting information


**DATA S1.** Table  reports the performance benchmarking of EnzyExtract. Text S1 lists the keywords used for literature search, and Text S2 describes the prompt template employed in EnzyExtract. Text S3 provides details on the extraction and comparison methods, while Text S4 outlines the dataset splitting strategies used in TurNuP, MESI, and DLKcat. Figure S1 shows the temperature and pH distribution in EnzyExtractDB; Figure S1 presents the distribution of *k*_cat_ and *K*_*m*_ values; Figure S3 illustrates the Morgan fingerprint diversity; and Figure S4 highlights the mutation diversity across the database.

**DATA S2.** Supporting Information.

## Data Availability

The raw data for EnzyExtractDB are available in the accompanying SI.zip file, which contains the following 10 folders: (1) dataset_223_EnzyExtract_BRENDA_manual_curation, providing the data underlying Table 1; (2) dataset_384_pipeline_benchmark_dataset, used to generate Table S1; (3) dataset_aligned_sequence_dataset, containing data aligned with external databases; (4) dataset_crude_database, comprising the raw output from the EnzyExtract pipeline; (5) dataset_enzyextract_brenda_correlation, including all datapoints utilized to plot the *k*_cat_ and *K*_*m*_ parity plot in Figure 3; (6) dataset_gpt4_finetuned, containing the PubMed article dataset used for fine‐tuning GPT‐4o‐mini; (7) dataset_OCR_dataset, which holds the data used to fine‐tune ResNet‐18; (8) dataset_retrain_kcat_predictor, providing the dataset used to retrain the kcat predictor; (9) EnzyExtractDB, containing a parquet format 176,463 datapoints of database; and (10) EnzyExtract‐main, which includes the source code. The EnzyExtract source code and EnzyExtractDB are also publicly available at https://github.com/ChemBioHTP/EnzyExtract, with an interactive Google Colab demo provided at https://colab.research.google.com/drive/1MwKSEZzLPNOseksRshbzkkFoO_cgJhva.
