# Supplementary material for: Finding the dark matter: Large language model‐based enzyme kinetic data extractor and its validation
Source: Protein Sci. 2025 Aug 15;34(9):e70251. doi: 10.1002/pro.70251 (PMC12355964; doi:10.1002/pro.70251)
Supplement: Supplementary file 1 — DATA S1. Table reports the performance benchmarking of EnzyExtract. Text S1 lists the keywords used for literature search, and Text S2 describes the prompt template employed in EnzyExtract. Text S3 provides details on the extraction and comparison methods, while Text S4 outlines the dataset splitting strategies used in TurNuP, MESI, and DLKcat. Figure S1 shows the temperature and pH distribution in EnzyExtractDB; Figure S1 presents the distribution of k cat and K m values; Figure S3 illustrates the Morgan fingerprint diversity; and Figure S4 highlights the mutation diversity across the database. [file PRO-34-e70251-s001.pdf]

Finding the Dark Matter: Large Language Model-based Enzyme Kinetic Data Extractor and Its Validation

Galen Wei[1†], Xinchun Ran[1†], Runeem AI-Abssi[1], Zhongyue Yang[1-5]

[1]*Department of Chemistry, Vanderbilt University, Nashville, Tennessee 37235, United States*

[2]*Center for Structural Biology, Vanderbilt University, Nashville, Tennessee 37235, United States*

[3]*Vanderbilt Institute of Chemical Biology, Vanderbilt University, Nashville, Tennessee 37235, United States*

[4]*Data Science Institute, Vanderbilt University, Nashville, Tennessee 37235, United States*

[5]*Department of Chemical and Biomolecular Engineering, Vanderbilt University, Nashville, Tennessee 37235, United States*

[†]Galen Wei and Xinchun Ran have contributed equally to this study.

Contents

Table S1 The performance benchmarking of EnzyExtract Page S2
Text S1 The keywords use for searching the articles Page S4
Text S2 The prompt of EnzyExtract Page S5
Text S3 The method details of extracted comparison Page S6
Text S4 Dataset splitting methods in TurNuP, MESI, and DLKcat Page S7
Figure S1 The temperatures, pH and EC first digit distribution of EnzyExtractDB Page S7
Figure S2 The k_{cat} and K_m distribution of EnzyExtractDB Page S8
Figure S3 The Morgan fingerprint diversity of EnzyExtractDB Page S9
Figure S4 The mutation diversity of EnzyExtractDB Page S10
Reference Page S12

Table S1 Detailed benchmarking of enzyme kinetics extraction across LLMs, OCR and table preprocessing variants, and input formatting (PDF vs. markdown). We evaluate the performance of EnzyExtract, GPT-4o-mini, GPT-4o (May 2024 version), and Claude 3.7 on k_{cat} and K_M extraction from 384 full-text publications (344 unique PMIDs). Table S1a benchmarks k_{cat} extraction using 1,909 ground-truth values. The full EnzyExtract pipeline—featuring OCR correction, TableTransformer parsing, and fine-tuned GPT-4o-mini—achieved the highest F1 score (0.83), with 1,348 true positives (TP), 334 false positives (FP), and 227 false negatives (FN), and maintained low magnitude error rates (2 values off by 60×, none by 3,600×). Removing table parsing reduced F1 to 0.69, while removing OCR yielded slightly improved recall (F1 = 0.84) but introduced instability in predictions. GPT-4o-mini (unfine-tuned) models underperformed (F1 = 0.69–0.70), with notable drops when table or OCR preprocessing was omitted. Claude 3.7 achieved strong precision (0.83) and an F1 of 0.80. The default GPT-4o (2024-05-13), which processes markdown-converted content rather than raw PDFs, achieved strong performance (F1 = 0.82) but occasionally introduced format-related artifacts. Table S1b benchmarks K_M extraction using 3,009 ground-truth values. EnzyExtract_full again achieved the highest balance (F1 = 0.80), while ablation of OCR or table preprocessing led to degraded accuracy (F1 = 0.63–0.70), with the no-OCR variant exhibiting the highest rate of magnitude errors (517 values off by ~100×). GPT-4o-mini showed lower F1 (0.54–0.68) and a high number of over- or underestimations. Claude 3.7 yielded the best precision overall (0.85) and an F1 of 0.81. The markdown-converted GPT-4o-05 model also performed well (F1 = 0.80), but like others, it suffered from higher numeric deviations. Across both tables, results confirm that each preprocessing step—OCR, table parsing, and input format fidelity (avoiding markdown artifacts)—significantly impacts extraction accuracy, and that domain-specific fine-tuning is essential for expert-level performance.

(a)

k_{cat} extraction	OCR preprocess	Table preprocess	num_pmids	TP	FP	FN	precision	recall	f1	accuracy	Off by 60	Off by 3600
EnzyExtract_full	TRUE	TRUE	327	1348	334	227	0.80	0.86	0.83	0.71	2	0
finetuned	TRUE	FALSE	328	1071	446	506	0.71	0.68	0.69	0.53	13	0
finetuned	FALSE	TRUE	325	1369	309	208	0.82	0.87	0.84	0.73	0	0
gpt-4o-mini-2024-07-18	TRUE	TRUE	315	1053	426	508	0.71	0.67	0.69	0.53	0	0
gpt-4o-mini-2024-07-18	TRUE	FALSE	322	827	449	735	0.65	0.53	0.58	0.41	25	0
gpt-4o-mini-2024-07-18	FALSE	TRUE	321	1050	365	525	0.74	0.67	0.70	0.54	8	0
gpt-4o-2024-05-13	TRUE	TRUE	318	1286	350	220	0.79	0.85	0.82	0.69	1	0
claude-3-7-sonnet-20250219	TRUE	TRUE	321	1153	240	337	0.83	0.77	0.80	0.67	3	0

(b)

K_M extraction	OCR preprocess?	Table preprocess?	num_pmids	TP	FP	FN	precision	recall	f1	accuracy	Off by 10^3	Off by 10^3
EnzyExtract_full	TRUE	TRUE	327	1992	535	482	0.79	0.81	0.80	0.66	129	0

finetuned	TRUE	FALSE	328	1676	667	783	0.72	0.68	0.70	0.54	180	0
finetuned	FALSE	TRUE	325	1597	973	881	0.62	0.64	0.63	0.46	517	0
gpt-4o-mini-2024-07-18	TRUE	TRUE	315	1525	501	939	0.75	0.62	0.68	0.51	151	0
gpt-4o-mini-2024-07-18	TRUE	FALSE	322	1294	396	1169	0.77	0.53	0.62	0.45	131	0
gpt-4o-mini-2024-07-18	FALSE	TRUE	321	1221	799	1262	0.60	0.49	0.54	0.37	483	0
gpt-4o-2024-05-13	TRUE	TRUE	318	1944	527	449	0.79	0.81	0.80	0.67	154	0
claude-3-7-sonnet-20250219	TRUE	TRUE	321	1819	324	534	0.85	0.77	0.81	0.68	46	0

Text S1 The Keywords used for searching the articles. To systematically construct a representative corpus of enzymology publications for training, fine-tuning, and human evaluation of the EnzyExtract pipeline, we performed large-scale literature mining using both Web of Science and OpenAlex databases. A comprehensive list of domain-specific keywords was curated to capture the diversity of reporting styles in enzyme kinetics literature, including both general terms (e.g.,

enzyme kinetics, enzyme catalysis) and specific phrases targeting kinetic constants, mutational studies, and directed evolution experiments (e.g., k_{cat}, mutation kinetics, site-saturation kinetics, NNK k_{cat}). In total, 21 unique search terms were applied across the two platforms (see the tables below for the breadkdown), resulting in thousands of potentially relevant hits (e.g., enzyme catalysis returned 47,931 records in Web of Science, and Michaelis, turnover retrieved 18,030 entries from OpenAlex[1]). The keywords and the number of returned records per query are listed in the accompanying table. This dual-platform strategy ensured comprehensive coverage of both classical enzymology literature and contemporary high-throughput mutational studies, enabling the creation of a high-quality, diverse dataset for both training and evaluation of our extraction models. By leveraging both keyword breadth and controlled specificity, we were able to maximize coverage while minimizing noise in the dataset curation process. These search terms laid the foundation for downstream manual curation and annotation efforts, ultimately supporting the construction of a 344-PMID benchmark dataset aligned with our gold-standard evaluation framework (see Table S1).

Web of Science keywords	Count
enzyme catalysis	47931
enzyme kinetics	45563
selectivity & enzyme	24116
enzyme rate constant	14039
mutant kinetics	13402
mutation kinetics	11942
wild type kinetics	10070
kinetic characterization enzyme	8990
mutagenesis kinetics	4790
directed evolution kinetics	3297
enzyme kcat	896
enantioselection & catalyzed	226
mutation kcat	179
site-saturation kinetics	165
directed evolution kcat	84
NNK kinetics	43
site-saturation kcat	11
NNK kcat	2

OpenAlex keywords	Count
Michaelis, cat	31680
Michaelis, kinetic characterization, enzyme	18980
Michaelis, turnover	18030
turnover km, enzyme	15030
kcat	14100
Michaelis, cat, kinetics, substrate	12980
Michaelis, cat, kinetics, enzyme	12900
cat km, characterization, substrate	9165
cat km, characterization, enzyme	8989
Michaelis, cat km	6024
cat km, characterisation, substrate	2193
cat km, characterisation, enzyme	1627

Text S2 The prompt of EnzyExtract. "You are a helpful and diligent assistant responsible for extracting K_m and k_{cat} data from tables from pdfs. For each data point, you extract the k_{cat} (turnover number), K_m (Michaelis constant), k_{cat}/K_m (catalytic efficiency), substrate, and descriptor. When extracting, you are primarily interested in k_{cat} with units time^-1, and Km with units of molarity. (ie. mM, nmol/mL, etc.) Therefore, you must exclude Vmax, specific activity, K_i, etc. k_{cat} and K_m should be formatted like so: "33 ± 0.3 s^-1" or "2.3 mM". Report the error if present. Keep original units and values (do not convert units), but attempt to clean up OCR errors. Be thorough and extract all data points present, including wild-type. The k_{cat} and K_m value must correspond to the descriptor. The descriptor and substrate fields must together contain all the information to uniquely identify the entry. The descriptor will most likely contain the enzyme, but it may also contain conditions like mutant code, organism, pH, temperature, etc. The substrate should be whatever corresponds to the K_m value. At the end, contextualize the data. Report all described enzymes, substrates,

mutants, organisms, temperatures, pHs, solvents, etc. Reference descriptor fragments verbatim. In the context, also include information common to all the entries in the table. For instance, if all the entries share the same enzyme and organism, report that in the context rather than in every descriptor. Before your final answer, you may write thoughts and comments, like observing which enzymes, substrates and conditions to report and how the table entries vary. Then, format your final answer like this example:


```yaml
data:
  - descriptor: wild-type cat-1
    substrate: H2O2
    kcat: 1 min^-1
    Km: null
    kcat/Km: null
  - descriptor: R190Q cat-1; 25°C
    substrate: H2O2
    kcat: 33 ± 0.3 s^-1
    Km: "2.3 mM"
    kcat/Km: null
  - descriptor: R203Q cat-1; (with NADPH); 25°C
    substrate: H2O2
    kcat: null
    Km: 9.9 ± 0.1 µM
    kcat/Km: 4.4 s^-1 mM^-1
```

~~~
context:
  enzymes:
    - fullname: catalase
      synonyms: cat-1
      mutants: wild-type; R190Q; R203Q
      organisms: Escherichia coli
  substrates:
    - fullname: hydrogen peroxide
      synonyms: H2O2
    - fullname: water
  temperatures: 25°C; 30°C
  pHs: 7.4
  other: NADPH
```”
~~~


Text S3 Similarity-based matching and accuracy definition for extracted kinetic parameters. To evaluate extraction performance, numeric kinetic values (k_{cat}, K_m) were matched to reference values with a strict tolerance of 0.0001%. Matching values were classified as true positives (TP), while unmatched values were classified as false positives (FP) or false negatives (FN); true negatives (TN) were not defined due to the open-ended nature of key-value space. To measure accuracy conditioned on correct enzyme–substrate identity, we implemented a similarity-based alignment scheme. Enzyme names and organisms were matched using the Indel similarity metric from the rapidfuzz library, with a match defined as ≥90% similarity for enzyme name. The

similarity score reflects the minimum edit distance normalized over the maximum string length. Additionally, enzyme EC numbers were used when available as exact-match keys. Substrates were aligned based on exact string match or identical SMILES strings, PubChem CID[2], or BRENDA[3] ID. Precision, recall, and F1-score were computed over this filtered set to quantify accuracy of kinetic parameter extraction given entity-level correspondence.

Text S4 Dataset Splitting Methods in TurNuP[4], MESI[5], and DLKcat[6]. We follow the dataset splitting methods reported in each original publication. TurNuP and MESI both employ enzyme-sequence-disjoint splits, where all data related to a given enzyme (defined by amino acid sequence) are confined to a single partition. TurNuP uses an 80/20 train-test split with fivefold cross-validation on the training set. MESI splits its dataset into 80% training, 10% validation, and 10% test. Both models stratify test enzymes based on global sequence identity to the training set using Needleman-Wunsch alignment, typically reporting results in four identity bins (0–40%, 40–80%, 80–99%, 99–100%). DLKcat, in contrast, applies a random split without ensuring sequence disjointness, resulting in high sequence redundancy across splits (e.g., 91% of test enzymes share ≥99% identity with training enzymes). DLKcat does not report performance by sequence similarity.

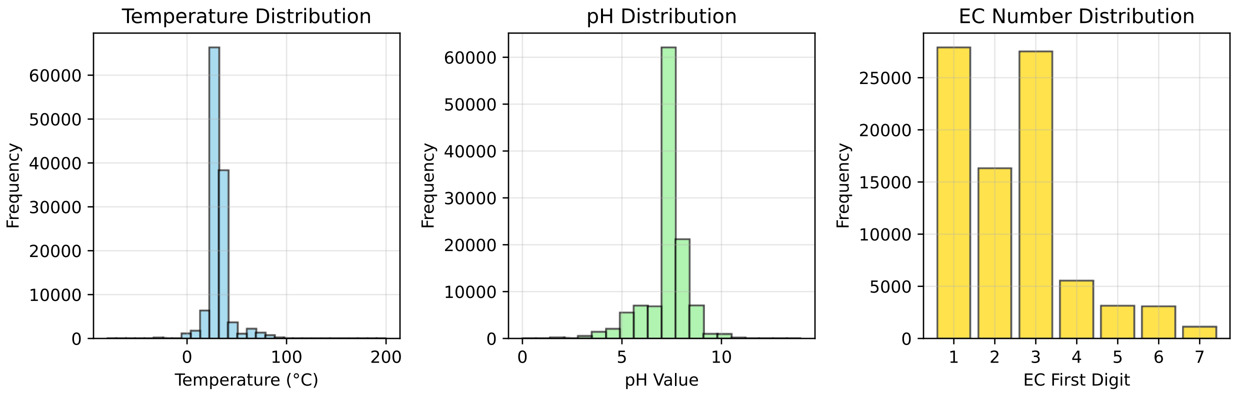

Figure S1 The temperature and pH distribution of EnzyExtractDB. Histogram plots representing the frequency distributions of optimal (left) temperatures and (right) pH values for enzymatic activity recorded in EnzyExtractDB. The temperature distribution is sharply centered between 30°C and 50°C, with a peak frequency of approximately 78,000 entries around 37°C, highlighting the predominance of mesophilic enzymes. The pH distribution follows a similarly narrow range, with the majority of entries concentrated between pH 6.0 and 8.0, and a maximum frequency of about 75,000 entries at pH 7.0, indicating a strong preference for near-neutral conditions. Both distributions underscore the dominance of enzymes adapted to physiological and standard laboratory environments. The EC number distribution is calculated by mapping enzyme names to EC numbers, discarding entries whose EC first digit cannot be uniquely determined. The EC Number distribution suggests that oxidoreductases (EC 1) and Hydrolases (EC 3) are well represented, while isomerases (EC 5), ligases (EC 6), and translocases (EC 7) remain underrepresented.

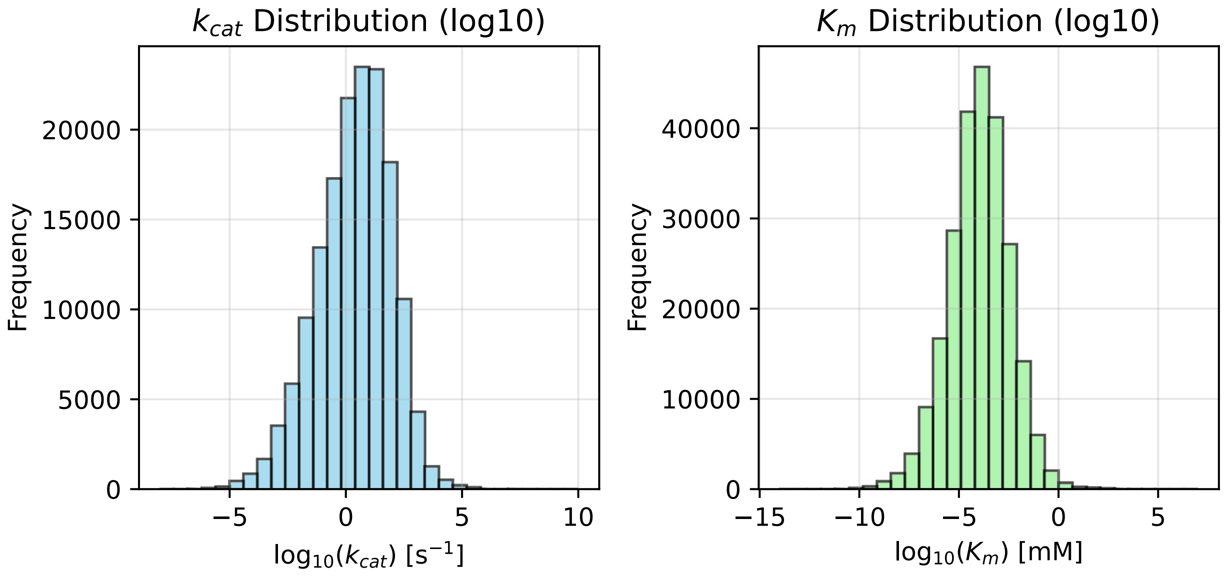

Figure S2 The k_{cat} and K_m distribution of EnzyExtractDB. Histogram plots showing the log10-transformed distributions of (left) k_{cat} values (s^{-1}) and (right) K_m values (mM) across enzyme entries in EnzyExtractDB. The log10(k_{cat}) values range from approximately –5 to 5, with a peak frequency of over 30,000 entries near log10(k_{cat}) ≈ 1.5, corresponding to a k_{cat} of about 30 s^{-1}. The log10(K_m) distribution spans from –10 to 5, peaking near log10(K_m) ≈ –1, equivalent to a K_M of 0.1 mM, with a maximum frequency of roughly 33,000 entries. These patterns reflect the central tendency of enzyme kinetics toward moderate catalytic efficiency and substrate affinity in biological systems.

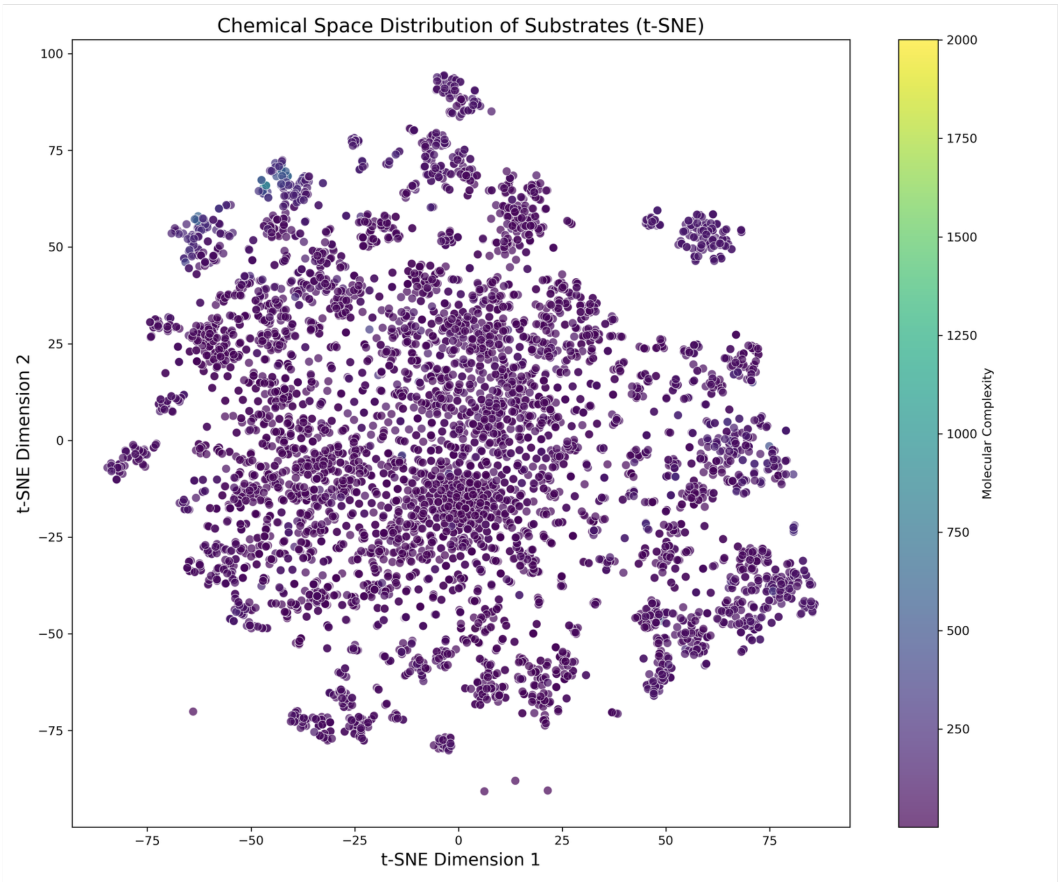

Figure S3 The Morgan fingerprint diversity of EnzyExtractDB. t-SNE (t-distributed stochastic neighbor embedding) visualization of the chemical space formed by 8,840 unique SMILES strings extracted from substrate molecules aligned to enzyme sequences via PubChem. Molecular similarity was encoded using Morgan fingerprints (radius = 2), and the resulting high-dimensional fingerprint vectors were projected onto two dimensions using t-SNE. Each point represents a unique substrate molecule, and color intensity corresponds to nearest-neighbor density, reflecting local molecular diversity. The plot highlights the broad and diverse chemical space of enzyme-associated compounds in EnzyExtractDB, with dense clusters indicating structurally similar substrates and sparser regions representing chemically distinct molecules.

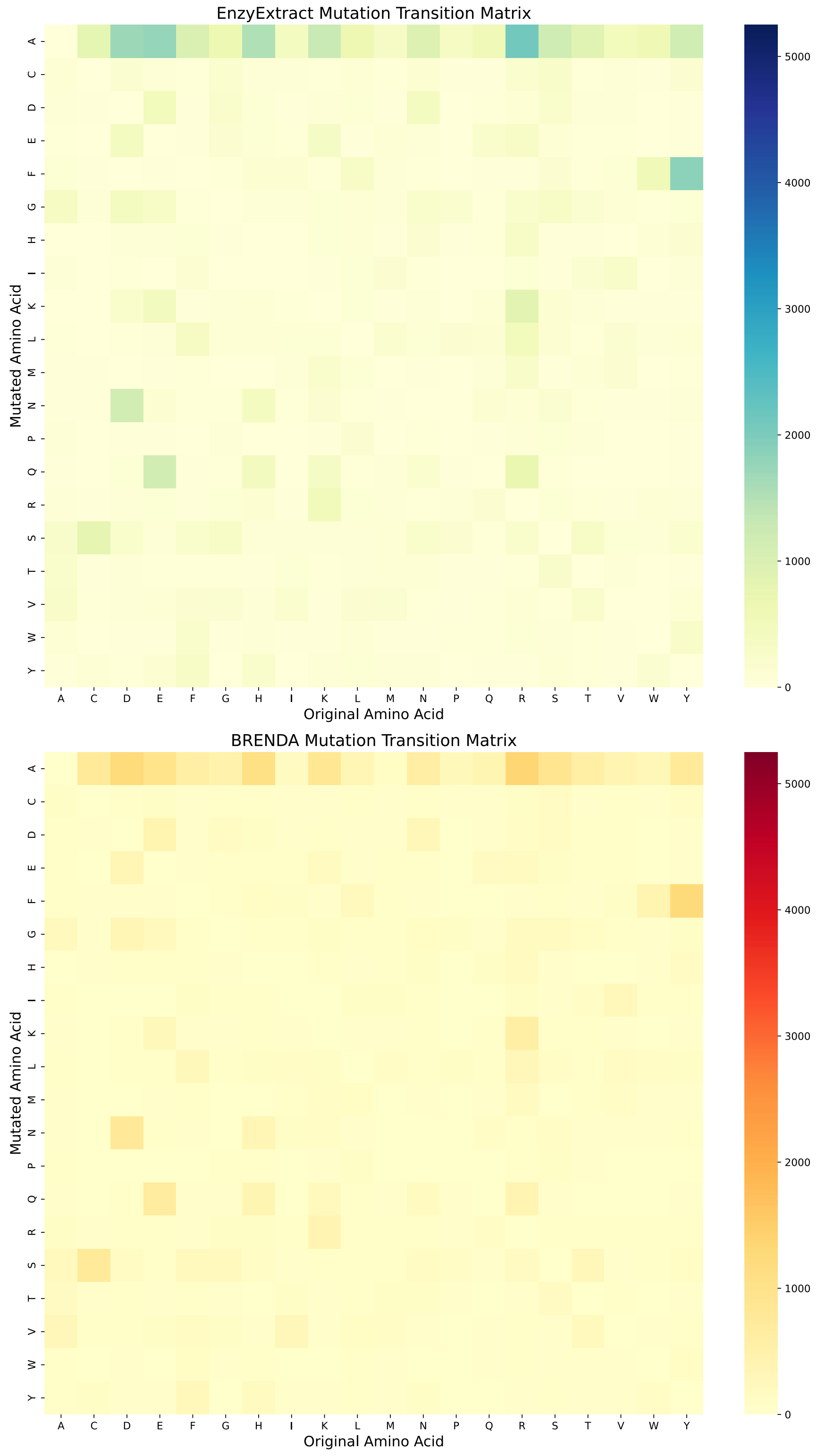

Figure S4 Comparative Analysis of Amino Acid Substitution Diversity in EnzyExtractDB and BRENDA. This figure presents heatmaps illustrating the distribution of observed amino acid

substitutions documented in EnzyExtractDB (top) and BRENDA (bottom). In each matrix, the x-axis denotes the wild-type (original) amino acid, while the y-axis indicates the corresponding mutant residue. Shading intensity reflects the relative frequency of each substitution event, with deeper blue indicating higher occurrence rates. In EnzyExtractDB, the five most frequently documented mutations—Ala>Arg (5.0%), Phe>Tyr (4.4%), Ala>Glu (4.2%), Ala>Asp (4.0%), and Ala>His (3.6%)—together account for 21.2% of all recorded substitution events. Correspondingly, BRENDA reports Ala>Arg (5.2%), Phe>Tyr (4.9%), Ala>Asp (4.8%), Ala>His (4.2%), and Ala>Glu (3.8%) as the most prevalent, comprising a combined 22.9% of total substitution cases. These percentages are calculated with respect to each database's total mutation records. The greater density of populated cells in EnzyExtractDB's transition matrix reflects increased coverage and diversity of experimentally characterized amino acid substitutions compared to BRENDA. Despite this improvement, the overall sparsity of both matrices indicates that only a small fraction of the theoretically possible mutational landscape is currently represented. The distribution of observed mutations is highly nonuniform, exemplifying substantial experimental and publication biases toward specific substitutions within the field of enzyme mutagenesis. Collectively, these observations underscore EnzyExtractDB's enhanced capacity for mutation coverage, while also highlighting the considerable unexplored expanse of the enzyme mutational landscape. The data further demonstrate that documented mutations are concentrated on a limited set of substitution types, providing valuable insight for future studies aimed at systematic and comprehensive exploration of protein sequence space

REFERENCE

(1) Priem, J.; Piwowar, H.; Orr, R. OpenAlex: A fully-open index of scholarly works, authors, venues, institutions, and concepts. *arXiv preprint arXiv:2205.01833* **2022**.
(2) Kim, S.; Chen, J.; Cheng, T.; Gindulyte, A.; He, J.; He, S.; Li, Q.; Shoemaker, B. A.; Thiessen, P. A.; Yu, B. PubChem 2023 update. *Nucleic acids research* **2023**, *51* (D1), D1373-D1380.
(3) Schomburg, I.; Chang, A.; Hofmann, O.; Ebeling, C.; Ehrentreich, F.; Schomburg, D. BRENDA: a resource for enzyme data and metabolic information. *Trends in biochemical sciences* **2002**, *27* (1), 54-56.
(4) Kroll, A.; Rousset, Y.; Hu, X.-P.; Liebrand, N. A.; Lercher, M. J. Turnover number predictions for kinetically uncharacterized enzymes using machine and deep learning. *Nature communications* **2023**, *14* (1), 4139.
(5) Nie, Z.; Zhang, H.; Jiang, H.; Liu, Y.; Huang, X.; Xu, F.; Tian, Y.; Chen, J.; Zhang, W.-B. Multi-purpose enzyme-substrate interaction prediction with progressive conditional deep learning. **2024**.
(6) Li, F.; Yuan, L.; Lu, H.; Li, G.; Chen, Y.; Engqvist, M. K.; Kerkhoven, E. J.; Nielsen, J. Deep learning-based k cat prediction enables improved enzyme-constrained model reconstruction. *Nature Catalysis* **2022**, *5* (8), 662-672.
